# Endometriosis as a Silent Emerging Malignant Threat in Young Women: A Case Report of Rare Rapid Transformation of Abdominal Wall Endometriosis Into Mixed High-Grade Serous and Clear Cell Carcinoma One Year Following Cesarean Section

**DOI:** 10.7759/cureus.114062

**Published:** 2026-08-06

**Authors:** May Hnin Oo, Ei Mon Mon Kyaw, Baian Alhindawi

**Affiliations:** 1 Obstetrics and Gynecology, Luton and Dunstable University Hospital, Bedfordshire Hospitals NHS Foundation Trust, Luton, GBR

**Keywords:** advanced ovarian cancer, cesarean scar endometriosis, diagnostic laparoscopy, high-grade serous ovarian carcinoma, ovarian clear cell carcinoma

## Abstract

Abdominal wall endometriosis most commonly occurs in cesarean section (CS) scars. Although malignant transformation is rare, clear cell carcinoma (CCC) is the most commonly reported histological subtype. We present an unusual case of rapid malignant transformation into mixed high-grade serous and CCC within one year following CS.

A 34-year-old para 1 woman presented with a two-month history of progressive shortness of breath, abdominal distension, abdominal pain, weight loss, and reduced appetite. Computed tomography (CT) demonstrated extensive peritoneal disease with bilateral ovarian lesions suspicious for advanced gynecological malignancy. She had a known history of endometriosis and had undergone a category 2 CS one year earlier, during which extensive pelvic endometriosis and decidualization were identified intraoperatively. Peritoneal biopsy performed at that time confirmed benign endometriosis without evidence of malignancy. Subsequent magnetic resonance imaging (MRI) demonstrated uterine scar endometriosis, abdominal wall scar endometriosis, and superficial ovarian endometriosis. Further investigations revealed malignant ascitic cytology consistent with metastatic high-grade serous carcinoma of gynecological origin. Diagnostic laparoscopy demonstrated extensive adhesions, ascites, diffuse peritoneal inflammatory disease, and complete obliteration of the pelvic anatomy. Histopathological analysis of biopsies obtained from the pelvic soft tissue, endometrioma cyst wall, and peritoneum confirmed mixed high-grade serous and CCC arising in a background of endometriosis.

Following multidisciplinary team discussion, the patient underwent extensive cytoreductive surgery, including total abdominal hysterectomy, bilateral salpingo-oophorectomy, peritonectomy, bilateral ureterolysis, pelvic lymph node excision, and infracolic omentectomy. She was subsequently diagnosed with stage IIIA2 ovarian mixed high-grade serous and CCC and commenced adjuvant chemotherapy with carboplatin and paclitaxel.

This case highlights the potential for rare, aggressive, and rapidly progressive malignant transformation of endometriosis, including transformation into mixed histological subtypes, in young women of reproductive age. Clinicians should maintain a high index of suspicion in patients with endometriosis who present with new systemic symptoms, ascites, or rapidly progressive pelvic or abdominal disease.

## Introduction

Endometriosis is an estrogen-dependent chronic inflammatory condition affecting approximately 5%-10% of women of reproductive age worldwide [1]. It is characterized by the ectopic growth of endometrial glands and stroma and commonly presents with dysmenorrhea, chronic pelvic pain, and infertility [1]. Although considered a benign condition, endometriosis exhibits several biological behaviors associated with malignancy, including local invasion, dissemination, recurrence, and resistance to apoptosis. These features have led to longstanding recognition of its malignant potential since it was first proposed by Sampson in 1925 [2].

Endometriosis-associated malignant transformation in abdominal surgical scars is an extremely rare but aggressive condition, most commonly occurring in previous cesarean section (CS) scars and typically demonstrating clear cell histology [3]. A Preferred Reporting Items for Systematic reviews and Meta-Analyses (PRISMA)-compliant systematic review by Mihailovici et al. reported that clear cell carcinoma (CCC) accounted for approximately 67% of published cases, with a median interval of approximately 19 years between surgery and the diagnosis of malignancy [3].

Abdominal wall endometriosis most commonly occurs in CS scars, and malignant transformation has increasingly been reported, most frequently as CCC. The average interval between CS and the diagnosis of malignancy is approximately 15 years [4]. Recent studies have provided molecular evidence that ovarian CCC, endometrioid carcinoma (EC), and seromucinous borderline tumors (SMBTs) may arise through the neoplastic transformation of ovarian endometriosis. These tumors are collectively referred to as endometriosis-related ovarian neoplasms (ERONs) [4].

High-grade serous carcinoma is considerably less common among endometriosis-associated malignancies. The coexistence of high-grade serous and clear cell components within the same endometriosis-associated malignancy is exceptionally rare, further emphasizing the unusual nature of the present case.

Our patient developed advanced-stage mixed high-grade serous and CCC within one year following CS, highlighting a rare, rapidly progressive, and aggressive disease course.

## Case presentation

A 34-year-old para 1 woman presented to the emergency department in October 2025 with a two-month history of progressive shortness of breath, generalized weakness, abdominal distension, abdominal pain, unintentional weight loss, and reduced appetite. On examination, she was hemodynamically stable; however, marked abdominal distension with massive ascites was noted.

Her past medical history was significant for known endometriosis and a large ovarian cyst identified in May 2024, measuring 78 × 43 × 46 mm. She subsequently underwent a category 2 CS in October 2024, during which extensive pelvic endometriosis and decidualization were identified intraoperatively. No endometrioma was visualized at the time of surgery. Peritoneal biopsy confirmed benign endometriosis without evidence of malignancy. Magnetic resonance imaging (MRI) performed in November 2024 demonstrated uterine scar endometriosis, abdominal wall scar endometriosis, and superficial ovarian endometriosis.

Initial laboratory investigations revealed an elevated cancer antigen 125 (CA-125) level of 257 U/mL. Other tumor markers, including carcinoembryonic antigen (CEA), lactate dehydrogenase (LDH), alpha-fetoprotein (AFP), and human chorionic gonadotropin (hCG), were within normal limits.

Computed tomography (CT) of the abdomen and pelvis demonstrated extensive abdominal and peritoneal disease with bilateral ovarian lesions suspicious for advanced gynecological malignancy. CT of the chest showed no evidence of pulmonary metastases. Pelvic MRI suggested deep infiltrating endometriosis complicated by pelvic inflammatory disease.

Diagnostic cytological examination of ascitic fluid, obtained in October 2025, demonstrated metastatic high-grade serous carcinoma of gynecological origin.

The patient subsequently underwent diagnostic laparoscopy in October 2025. Intraoperative findings demonstrated extensive inflammatory and adhesive disease with complete obliteration of the normal pelvic anatomy. A large volume of ascites was present.

**Figure 1:**
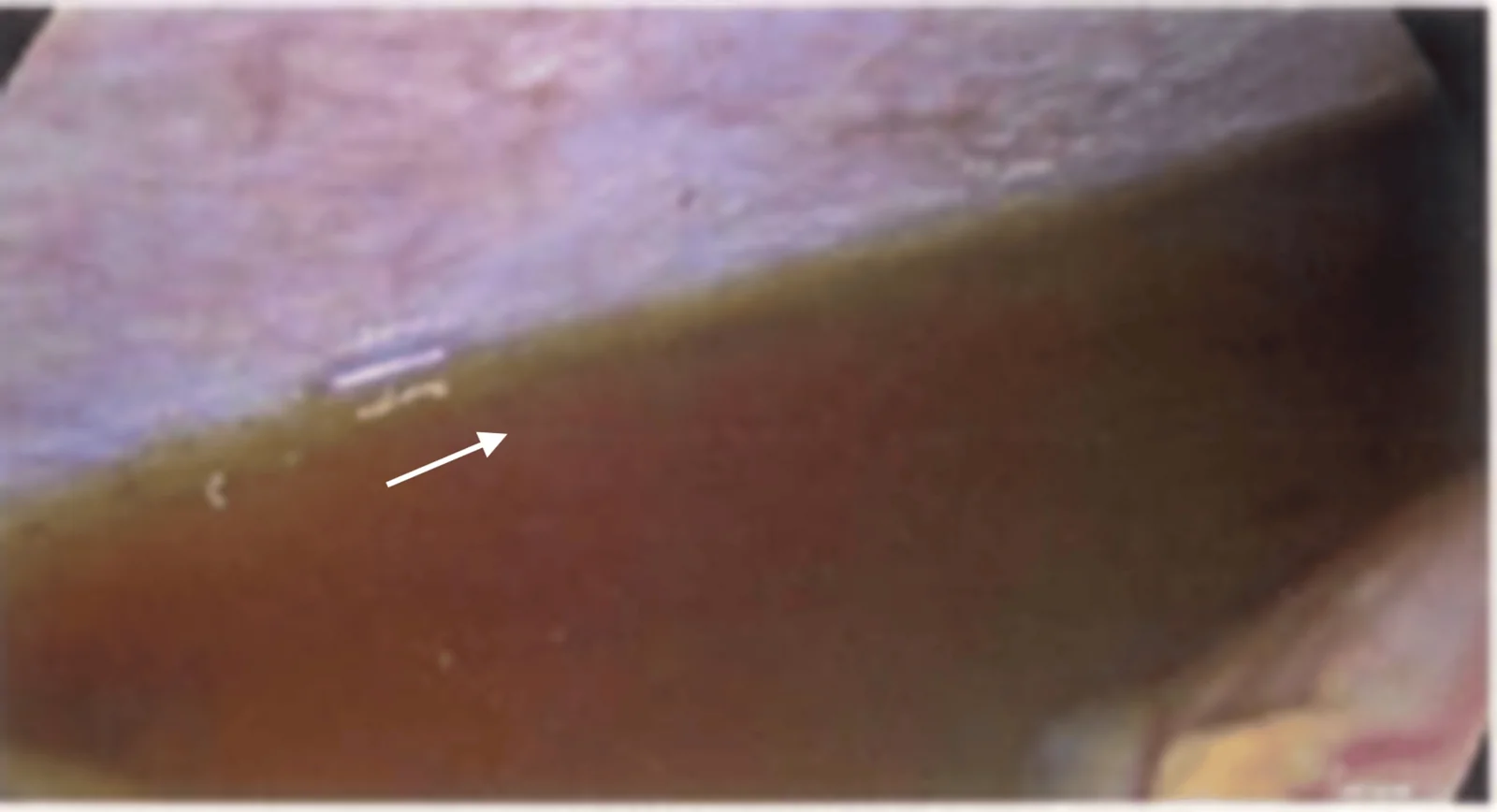
Laparoscopic view demonstrating significant ascites within the peritoneal cavity (white arrow)

As shown in Figure 1, diagnostic laparoscopy demonstrated a large volume of ascites within the peritoneal cavity, raising suspicion for advanced intra-abdominal malignancy.

Dense adhesions involving the bowel loops, pelvic sidewalls, bladder, uterus, and adnexal structures were identified. The pelvis was completely obliterated by dense bowel adhesions involving the pelvic sidewalls and anterior abdominal wall. The uterus was adherent to the anterior abdominal wall, while the bladder was densely adherent to both the uterus and the anterior abdominal wall. The bilateral adnexal structures were adherent posteriorly within the ovarian fossae and the pouch of Douglas.

**Figure 2:**
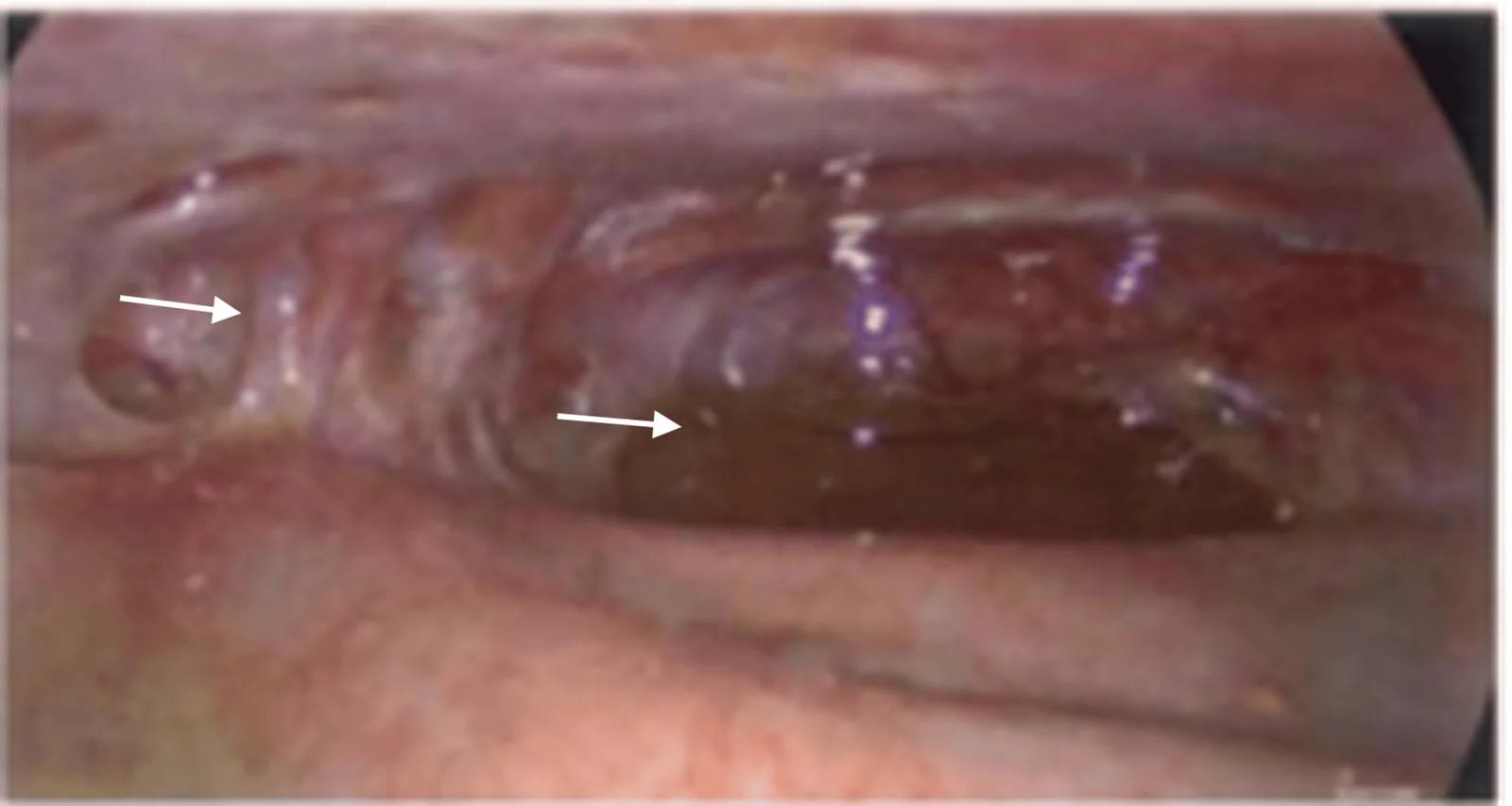
Intraoperative laparoscopic image showing extensive dense pelvic adhesions and ascitic fluid within the peritoneal cavity (white arrows)

As shown in Figure 2, diagnostic laparoscopy demonstrated extensive dense pelvic adhesions resulting in complete obliteration of the pelvic anatomy, with associated ascitic fluid within the peritoneal cavity.

**Figure 3:**
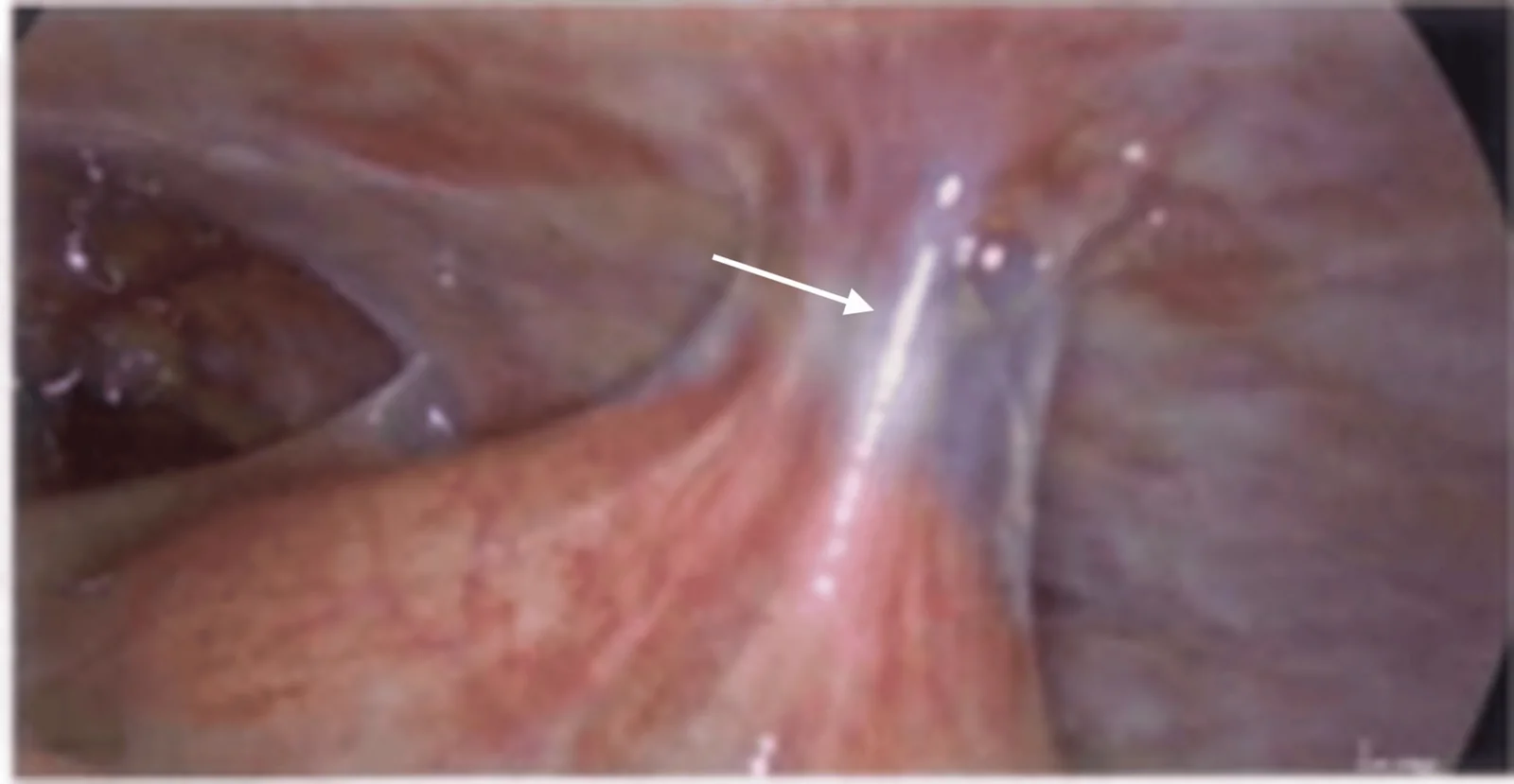
Laparoscopic view demonstrating adhesions between the omentum and anterior abdominal wall Figure 3. Dense adhesions between the omentum and the anterior abdominal wall (white arrow).

As shown in Figure 3, diagnostic laparoscopy demonstrated dense adhesions between the omentum and the anterior abdominal wall.

Diffuse peritoneal inflammatory changes and atypical miliary-type deposits were also noted, raising suspicion for advanced malignancy as well as alternative differential diagnoses, including tuberculous peritonitis. Interestingly, despite the extensive intra-abdominal disease, classical bulky omental caking was absent. Instead, predominantly thin, filmy adhesions overlying the bowel loops and diffuse inflammatory changes were observed.

**Figure 4:**
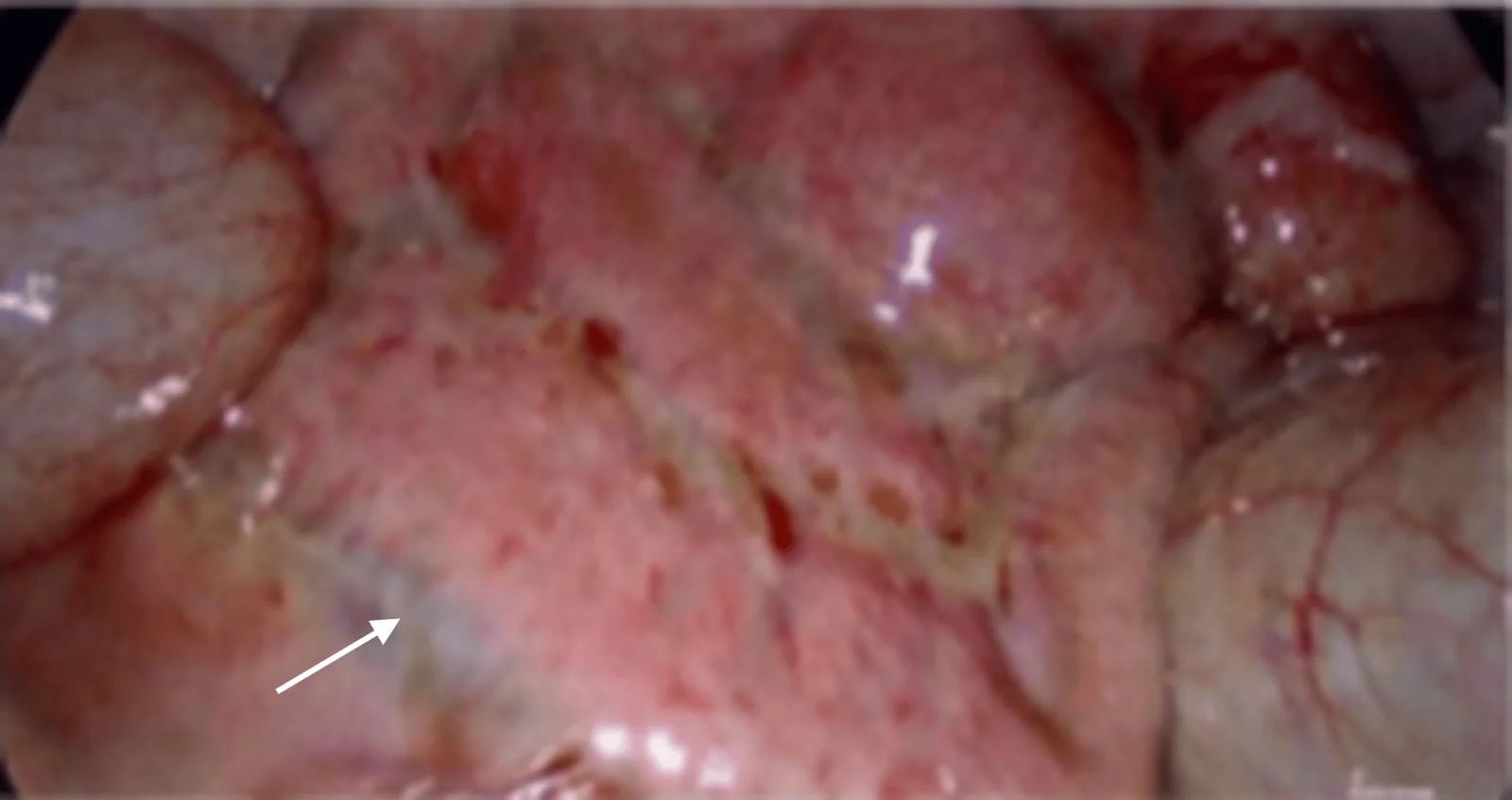
Laparoscopic image demonstrating thin filmy adhesions over the bowel with atypical miliary deposits (white arrow)

As shown in Figure 4, diagnostic laparoscopy demonstrated thin, filmy adhesions overlying the bowel loops, together with atypical miliary-type peritoneal deposits.

Biopsies were obtained from the pelvic soft tissue, endometrioma cyst wall, right and left iliac fossae peritoneum, and the left uterovesical fold. A small bladder injury occurred intraoperatively and was repaired by the urology team, with an uncomplicated postoperative recovery confirmed by CT urogram.

Histopathological examination confirmed mixed high-grade serous and CCC arising in a background of endometriosis. Ascitic fluid cytology demonstrated metastatic high-grade serous carcinoma of gynecological origin.

Following multidisciplinary team discussion, the patient was diagnosed with stage IIIA2 mixed high-grade serous and CCC. Tumor genomic profiling demonstrated homologous recombination deficiency (HRD)-negative disease with mismatch repair (MMR)-proficient status.

The patient was referred to a tertiary gynecological oncology center and underwent extensive cytoreductive surgery in December 2025. The procedure included a midline laparotomy with drainage of 1.5 L of clear ascitic fluid, extensive adhesiolysis, total abdominal hysterectomy with bilateral salpingo-oophorectomy, pelvic and bladder peritonectomy, bilateral ureterolysis, excision of a bulky left external iliac lymph node, and infracolic omentectomy.

Operative findings included markedly distorted pelvic anatomy, bilateral adnexal masses adherent to the pelvic sidewalls, extensive pelvic and bladder peritoneal disease, and matted loops of inflamed small bowel adherent to the anterior abdominal wall. The pelvis was completely obliterated by dense adhesions involving both the small and large bowel. The omentum appeared grossly contracted but smooth, without the typical appearance of omental caking. A solitary enlarged left external iliac lymph node was identified.

The postoperative recovery was uncomplicated. The patient commenced adjuvant chemotherapy with carboplatin and paclitaxel in January 2026. Follow-up imaging, reviewed at the local multidisciplinary team meeting in January 2026, demonstrated increased accumulation of pleural fluid within the left loculated subdiaphragmatic pouch, gastrohepatic pouch, superior recess of the lesser sac, and the anterior and posterior pelvic peritoneal pouches. These findings were considered more consistent with an inflammatory process than with definite disease progression. Continuation of chemotherapy, with interval re-imaging following completion of treatment, was recommended.

At the time of writing, the patient remained under ongoing oncological follow-up and was awaiting subsequent chemotherapy cycles.

Table 1 presents the chronological timeline summarizing the patient’s clinical presentation, imaging findings, pathological diagnosis, surgical management, and oncological treatment.

**Table 1:** Timeline of the patient’s clinical presentation, investigations, diagnosis, surgery, and treatment

Date	Clinical events and findings
May 2024 (anomaly scan)	Right endometriotic cyst measuring 78 x 43 x 46 mm
October 2024	Category 2 cesarean section: extensive pelvic endometriosis with decidualization. Peritoneal biopsy: benign endometriosis with no evidence of malignancy
November 2024	Pelvic MRI: endometriosis involving the uterine cesarean scar and anterior abdominal wall, with superficial ovarian endometriosis
October 2025	Presented to A&E with shortness of breath, progressive abdominal distension and pain, reduced appetite, and weight loss, significant ascites. Serum CA-125: 257 U/mL. CT: extensive peritoneal disease, ascites, and bilateral ovarian lesions, raising suspicion of advanced gynecological malignancy. Ascitic fluid cytology: metastatic high-grade serous carcinoma of gynecological origin. Diagnostic laparoscopy: extensive ascites, dense pelvic adhesions with obliteration of the pelvic anatomy, and atypical miliary deposits. Histological examination of laparoscopic biopsies: mixed high-grade serous and clear cell carcinoma
December 2025	Extensive cytoreductive surgery
January 2026	Adjuvant chemotherapy with carboplatin and paclitaxel

## Discussion

Malignant transformation of endometriosis is an uncommon but increasingly recognized phenomenon, most frequently involving ovarian endometriosis and histological subtypes, such as CCC and endometrioid carcinoma [1,2]. Extra-ovarian malignant transformation, particularly arising from abdominal wall or cesarean scar endometriosis, remains rare [3,4].

The diagnosis of malignant transformation arising from endometriosis has traditionally been assessed using the Sampson and Scott criteria. Sampson proposed that endometriosis and carcinoma should coexist within the same lesion, that the histological appearance should be compatible with an endometrial origin, and that another primary tumor should be excluded [2]. Scott subsequently added the requirement for histological evidence of a direct transition from benign endometriosis to malignant tissue, as summarized in a later systematic review [3].

In the present case, previously confirmed benign endometriosis and the subsequent development of mixed high-grade serous and CCC support an association between endometriosis and these tumors. However, a direct histological transition from benign or atypical endometriosis to invasive carcinoma was not demonstrated, and the precise primary anatomical site could not be established. Therefore, although the findings support an endometriosis-associated carcinoma, they do not conclusively fulfil all the criteria for proven malignant transformation.

CCC arising from abdominal wall endometriosis generally carries a poor prognosis because of its aggressive biological behavior, high recurrence rate, and tendency to present at an advanced stage. The reported median survival is approximately 30 months [5]. Owing to the rarity of this condition, the optimal roles of cytoreductive surgery, adjuvant chemotherapy, radiotherapy, and surveillance remain unclear; consequently, management is often individualized within a multidisciplinary setting [5].

Ferrari et al. reviewed reported cases of CCC arising from abdominal wall endometriosis and demonstrated that malignant transformation typically occurs many years after the initial surgical procedure, with a mean interval of approximately 15 years following CS [4]. In contrast, our patient demonstrated an unusually rapid and aggressive clinical course, with the development of advanced-stage malignancy within one year of CS and after a relatively short duration of symptoms. This case therefore highlights an important clinical concern that malignant transformation of scar endometriosis may not always follow the prolonged latent period traditionally described in the literature.

Despite the relatively short duration of symptoms, our patient had already developed extensive intra-abdominal disease at presentation, suggesting highly aggressive tumor biology. Previous studies have demonstrated a significant association between endometriosis and ovarian malignancy, particularly ovarian CCC and endometrioid ovarian carcinoma [6]. Proposed mechanisms underlying malignant transformation include chronic inflammation, oxidative stress, hormonal stimulation, immune dysregulation, and the accumulation of cancer-associated genetic alterations, including ARID1A and PIK3CA mutations [7]. These molecular and biological characteristics may have contributed to the aggressive clinical behavior and rapid disease progression observed in our patient.

In our case, diagnostic laparoscopy played a pivotal role in establishing the diagnosis. Preoperative imaging findings were complex and initially raised differential diagnoses including deep infiltrating endometriosis, pelvic inflammatory disease, and tuberculous peritonitis. However, laparoscopic assessment enabled direct visualization of extensive peritoneal disease, dense adhesions, complete obliteration of the pelvic anatomy, and atypical miliary deposits, facilitating targeted biopsies for definitive histopathological diagnosis.

Interestingly, despite extensive intra-abdominal disease, laparoscopic assessment did not demonstrate the classical appearance of dense omental caking typically associated with advanced ovarian malignancy. Instead, predominantly thin, filmy adhesions overlying the bowel loops and diffuse inflammatory changes were observed. These atypical intraoperative findings further contributed to the diagnostic complexity and highlight that endometriosis-associated malignancy may mimic severe inflammatory or infective intra-abdominal pathology.

Complete cytoreductive surgery remains the cornerstone of management for advanced endometriosis-associated ovarian malignancy. Our patient underwent extensive cytoreductive surgery followed by adjuvant carboplatin and paclitaxel chemotherapy and remains under ongoing oncological surveillance. However, owing to the rarity of malignant transformation arising from abdominal wall endometriosis, evidence regarding optimal treatment strategies, surveillance protocols, and long-term outcomes remains limited.

This case contributes to the growing body of literature on aggressive endometriosis-associated malignancy and emphasizes the importance of early recognition, multidisciplinary management, and heightened clinical awareness of atypical presentations in women with known endometriosis. Rapidly progressive symptoms, enlarging scar lesions, ascites, or atypical radiological findings should prompt consideration of malignant transformation, even in patients without a longstanding history of scar endometriosis.

## Conclusions

This case suggests an exceptionally rapid malignant transformation associated with abdominal wall endometriosis, resulting in mixed high-grade serous and CCC within one year following CS. However, as the precise anatomical origin and a direct histological transition from benign endometriosis to carcinoma were not demonstrated, a previously occult or unsampled malignancy cannot be excluded. Although malignant transformation of endometriosis remains rare, clinicians should maintain a high index of suspicion in women with known endometriosis who present with new systemic symptoms, ascites, rapidly progressive pelvic disease, or atypical radiological findings.

Importantly, this case highlights that advanced endometriosis-associated malignancy may present with atypical inflammatory laparoscopic appearances rather than the classical bulky omental disease typically associated with advanced ovarian malignancy, thereby creating significant diagnostic challenges. Diagnostic laparoscopy can therefore play an important role in selected complex cases by enabling direct visualization, targeted tissue sampling, timely histopathological diagnosis, and multidisciplinary treatment planning. Early recognition and prompt multidisciplinary oncological management remain essential for these rare and aggressive malignancies.
